# *QuickStats***:** Number of Deaths from 10 Leading Causes,* by Sex — National Vital Statistics System, United States, 2015

**DOI:** 10.15585/mmwr.mm6615a8

**Published:** 2017-04-21

**Figure:**
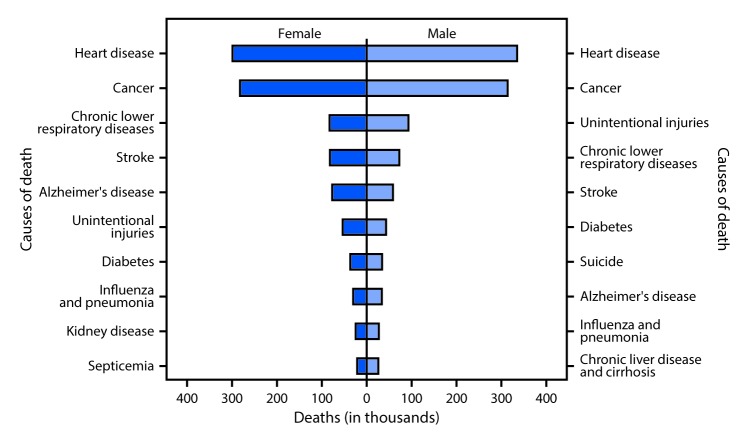
In 2015, a total of 1,339,226 deaths among females and 1,373,404 deaths among males occurred. Heart disease and cancer were the top two causes of death for both females and males; other leading causes varied in rank by sex. The 10 leading causes of death accounted for approximately three-quarters of all deaths.

**Source:** National Vital Statistics System. Underlying cause of death data, 2015. https://wonder.cdc.gov/ucd-icd10.html.

